# Adaptive, Clinically Guided Multimodal Therapy with Supportive Drug Sensitivity Testing in a Dog with Hepatic Neuroendocrine Carcinoma: A Case Report

**DOI:** 10.3390/ani16040646

**Published:** 2026-02-17

**Authors:** Kyu-Duk Yeon, Jin-Young Choi, Ji-Hyeok Seo, Kieun Bae, Joong-Yeon Choi, Chang-Hun Moon, Kyong-Ah Yoon, Jung-Hyun Kim

**Affiliations:** 1 Department of Veterinary Internal Medicine, College of Veterinary Medicine, Konkuk University, Seoul 05029, Republic of Korea; gundam223@naver.com; 2 SNC Animal Medical Center, 416 Nonhyeon-ro, Gangnam-gu, Seoul 06134, Republic of Korea; aditee67@naver.com (J.-Y.C.); nurong3000@hanmail.net (J.-H.S.); choi.joongyeon@sncamc.kr (J.-Y.C.); doorwindow@sncamc.kr (C.-H.M.); 3 KU Animal Cancer Center, Konkuk University Veterinary Medical Teaching Hospital, 120, Neungdong-ro, Gwangjin-gu, Seoul 05029, Republic of Korea; kieun86@konkuk.ac.kr (K.B.); kayoon@konkuk.ac.kr (K.-A.Y.); 4 Department of Veterinary Biochemistry, College of Veterinary Medicine, Konkuk University, Seoul 05029, Republic of Korea

**Keywords:** drug sensitivity test, hepatic neuroendocrine carcinoma, adaptive chemotherapy, toceranib phosphate, natural killer cell activator

## Abstract

Hepatic neuroendocrine carcinoma (NEC) in dogs is a rare malignancy with limited therapeutic guidance and no established standard of care. This report describes an adaptive, clinically guided multimodal treatment approach in a dog with advanced hepatic NEC with regional lymph node involvement. Sequential systemic therapies—including doxorubicin, mitoxantrone with lomustine and prednisolone, and subsequently toceranib—were administered based on clinical response assessment using imaging (VCOG RECIST), hematologic monitoring, and quality-of-life evaluation. Ex vivo drug sensitivity testing (DST) was performed to provide functional reference information but was interpreted as supportive rather than predictive. Notably, discordance was observed between strong in vitro sensitivity to doxorubicin and early clinical progression, underscoring the limitations of monoculture-based assays in recapitulating in vivo tumor biology. Sustained stable disease was observed following transition to toceranib with continued adjunct immunomodulatory therapy; however, the independent contribution of each treatment component cannot be determined. This case highlights the feasibility of iterative treatment refinement in rare canine malignancies and emphasizes that DST findings should be integrated cautiously within a broader clinical decision-making framework.

## Simple Summary

This case describes a dog with advanced hepatic neuroendocrine carcinoma characterized by multifocal intrahepatic lesions and regional lymph node metastasis, for which no standardized systemic protocol has been established. An ex vivo drug sensitivity assay was performed at diagnosis as supportive, hypothesis-generating information to complement clinical decision-making. Systemic therapy was adapted sequentially based on imaging response, tolerability, and quality-of-life considerations, including doxorubicin followed by mitoxantrone with lomustine and subsequently toceranib. A cytokine-based NK cell activator was administered as an adjunct during the treatment course; however, its independent clinical contribution could not be determined in the absence of immune or pharmacodynamic monitoring. Despite early discordance between in vitro findings and in vivo response, protocol transitions achieved sustained clinical stability with acceptable tolerability, highlighting the value of response-driven treatment adaptation in rare canine hepatic malignancies.

## 1. Introduction

Hepatic neuroendocrine carcinoma (NEC) is a very rare tumor in dogs, and most cases are diagnosed at an advanced stage with multifocal hepatic involvement and regional lymph node metastasis [1,2]. The prognosis is poor, with some reports describing rapid clinical deterioration within days of diagnosis, and no standardized therapeutic protocol has been established to date [1,2]. Although chemotherapeutic approaches using doxorubicin or toceranib have been reported in a limited number of cases, the consistency and reproducibility of treatment responses remain limited, and prognosis varies widely depending on individual biological characteristics [3,4].

Given the absence of a standard treatment and the potential for rapid disease progression, there is a clinical need for strategies that can provide supportive information regarding potential patient-specific therapeutic responsiveness while minimizing toxicity and maintaining treatment continuity. Functional ex vivo drug response testing has gained attention in human oncology for enabling personalized treatment selection in various cancer types, including rare tumors [5], and its potential application in veterinary oncology is also being increasingly recognized.

This case demonstrates the practical applicability of an adaptive, response-driven therapeutic strategy for canine hepatic NEC in a real-world clinical setting, while illustrating how DST may serve as supportive functional information when integrated with longitudinal clinical monitoring.

## 2. Case Description

**Figure 1:**
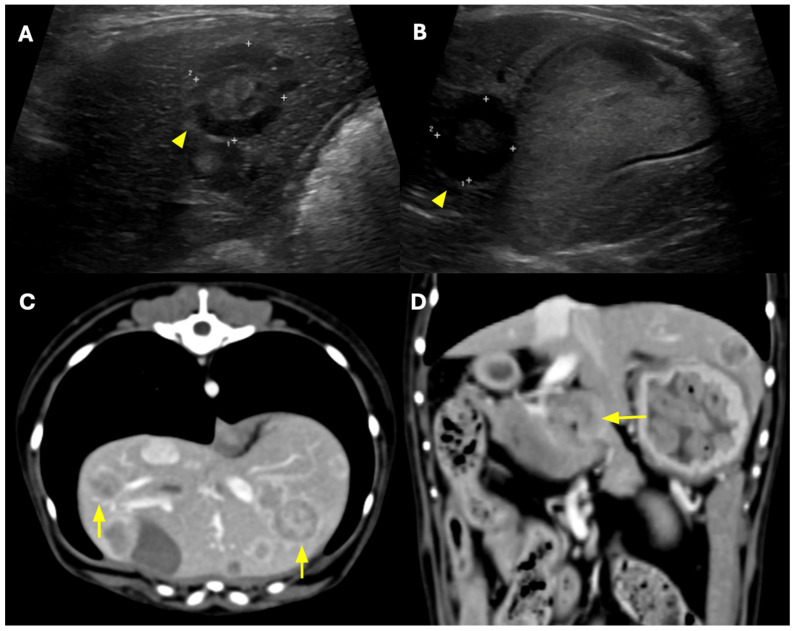
Ultrasonographic and CT findings of hepatic neuroendocrine carcinoma in a dog. (**A**,**B**) Ultrasonography revealed multiple hepatic nodules with a target-sign appearance (yellow arrowheads). Two representative nodules were selected for treatment monitoring: Mass 1 (**A**), adjacent to the stomach, and Mass 2 (**B**), adjacent to the gallbladder. Gallbladder sludge was also noted. (**C**) Transverse CT image in the portal phase showing multiple hepatic nodules (yellow arrows), measuring approximately 6–15 mm in diameter and exhibiting rim enhancement, with some containing internal fluid attenuation. (**D**) Coronal CT image demonstrating hepatic nodules and regional lymphadenopathy (yellow arrows).

A 7-year-old spayed female Maltese presented for a routine health check. The patient exhibited no apparent clinical signs, remained alert, and had normal vital parameters, including body temperature, heart rate, and blood pressure. Abdominal ultrasonography revealed multiple, well-demarcated hepatic nodules distributed throughout the liver. The nodules exhibited a target-like appearance, characterized by a hypoechoic peripheral rim and a relatively hyperechoic central region. Several lesions showed heterogeneous internal echogenicity with anechoic to hypoechoic areas suggestive of cystic or necrotic changes, raising suspicion for a primary hepatic neoplasm (Figure 1A,B). On subsequent computed tomography (CT), the hepatic nodules exhibited marked peripheral rim enhancement, and some lesions contained areas of fluid attenuation within their cores (Figure 1C,D).

In addition, a 16 × 6.4 × 10.5 mm mass with heterogeneous contrast enhancement was identified at the hepatic lymph node region, and the pancreatic lymph node was also enlarged with heterogeneous enhancement, raising suspicion of regional nodal metastasis. Accordingly, surgical excision of a distal hepatic nodule was performed together with biopsy of the adjacent hepatic hilar lymph node to assess metastatic involvement. Histopathological evaluation of the lymph node revealed morphological features consistent with neuroendocrine differentiation, confirming regional lymph node metastasis. Based on the combined imaging and pathological findings, the disease was characterized as multifocal intrahepatic lesions with regional lymph node metastasis and no evidence of distant metastasis. As no standardized TNM staging system has been formally established for canine hepatic neuroendocrine carcinoma, the extent of disease is described descriptively rather than assigned to a specific stage category.

To characterize the nature of the tumor, a Tru-cut biopsy was performed under sedation. However, the Tru-cut specimen provided insufficient information to clearly differentiate between a neuroendocrine tumor and carcinoma, and the amount of tissue obtained was inadequate for drug sensitivity testing (DST). In addition, histopathological evaluation alone was insufficient to establish a definitive diagnosis or determine an appropriate therapeutic plan. Therefore, to obtain a conclusive diagnosis and secure sufficient tissue for DST, surgical excision of a 1.0 × 1.0 × 1.0 cm mass located at the distal portion of the left lateral hepatic lobe was performed (Supplementary Figure S1). The resected tissue was divided, with one portion submitted for histopathological examination and the other for DST.

**Figure 2:**
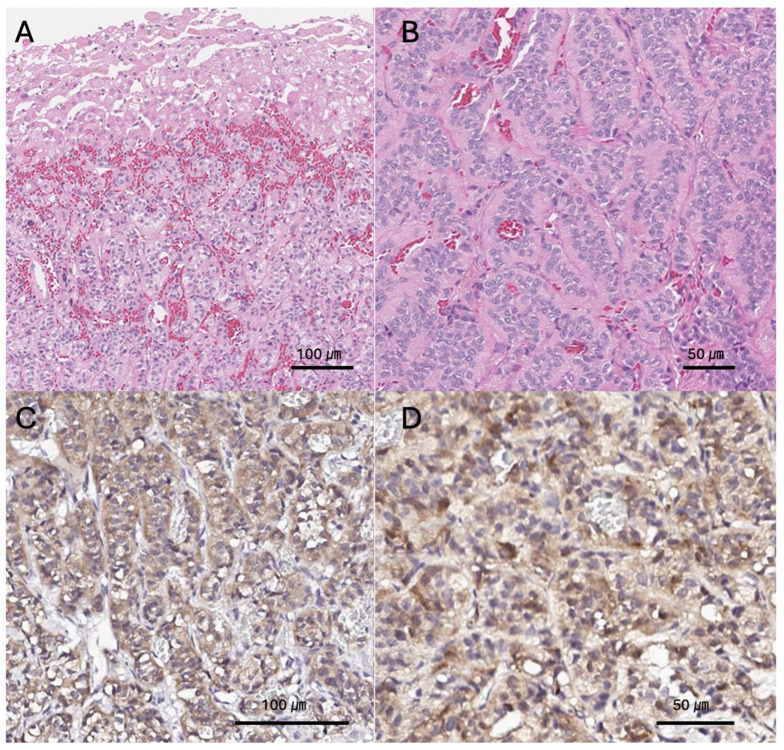
Histopathological and immunohistochemical features of hepatic neuroendocrine carcinoma. (**A**) Low-power H&E staining (original magnification ×200) shows tumor cells forming sheets and solid nests separated by fibrous septa (scale bar = 100 μm). (**B**) High-power H&E image (original magnification ×400) demonstrates rosette-like structures and perpendicular palisading of tumor cells along basement membranes. The cells exhibit prominent granular eosinophilic cytoplasm (scale bar = 50 μm). (**C**) Immunohistochemistry for NSE (original magnification ×300) reveals strong cytoplasmic positivity in tumor cells (scale bar = 100 μm). (**D**) Chromogranin A staining (original magnification ×600) shows diffuse and intense cytoplasmic positivity, confirming neuroendocrine differentiation (scale bar = 50 μm).

Histopathological examination of the resected tissue using hematoxylin and eosin (H&E) staining revealed neoplastic cells arranged in plates (sheets) or solid nests separated by fibrous septa (Figure 2A,B). The tumor exhibited rosette-like structures and perpendicular alignment of cells along the basement membrane, and the cytoplasm showed distinct granular eosinophilic characteristics. To refine the diagnosis, immunohistochemical staining was performed, which demonstrated strong positive immunoreactivity for neuron-specific enolase (NSE) and chromogranin A, confirming neuroendocrine differentiation (Figure 2C,D). Considering these morphological and immunohistochemical features, together with the presence of multifocal hepatic lesions and suspected lymph node metastasis, the tumor was diagnosed as an advanced primary hepatic neuroendocrine carcinoma with regional metastasis.

### Ex Vivo Drug Sensitivity Testing (DST)

To support therapeutic decision-making in a rare tumor type lacking standardized treatment protocols, DST was performed using patient-derived tumor cells obtained from the surgically excised hepatic nodule. Tumor tissue was mechanically minced and enzymatically dissociated, and isolated cells were cultured in Advanced DMEM/F12 medium supplemented with 10% fetal bovine serum, GlutaMAX™, Zellshield, and 1 M HEPES at 37 °C in a 5% CO_2_ atmosphere. Cell viability was confirmed using trypan blue exclusion prior to seeding, and only viable cells were plated at the indicated density. Cells were tested at passage 0 after a short attachment period to minimize culture-induced selection bias. For drug screening, cells were seeded in 96-well plates (1.5 × 10^4^ cells/well) and exposed to a panel of anticancer agents across concentrations ranging from 5 to 100 μM. Carboplatin, cyclophosphamide, lomustine (CCNU), and vincristine were tested at concentrations up to a maximum of 100 μM. After 24 h, cell viability was assessed using the CellTiter-Glo^®^ luminescent assay. Drug sensitivity was primarily interpreted based on relative dose–response patterns rather than formal IC50-driven ranking, given the single-case design and absence of biological replicates.

Drug responsiveness was interpreted descriptively based on observed dose–response patterns in cell viability under the tested conditions, without applying predefined quantitative thresholds. Doxorubicin, mitoxantrone, and toceranib were associated with concentration-dependent reductions in cell viability, whereas vincristine demonstrated variable effects across tested concentrations. Carboplatin, cyclophosphamide, and chlorambucil exhibited minimal measurable activity within the evaluated concentration ranges. These findings were considered exploratory and were used as supportive functional information rather than as a definitive ranking of therapeutic efficacy. Lomustine (CCNU) exhibited reductions in viability primarily at higher tested concentrations. Because cyclophosphamide requires hepatic metabolic activation, its in vitro assessment may not fully reflect in vivo efficacy; therefore, this result was interpreted with caution. In addition, the clinical relevance of lomustine effects observed only at supraphysiologic concentrations was considered limited.

All experiments were performed in technical triplicates (*n* = 3 wells per condition), and results are presented as mean ± SD. As this study represents a single-patient case, biological replicates were not available, and the DST findings should therefore be interpreted as supportive functional information rather than statistically powered comparative data. Accordingly, formal statistical correlation between ex vivo drug sensitivity and subsequent clinical response was not performed, and concordance was interpreted qualitatively in the Discussion.

Because no tumor cell purification or lineage-specific validation was performed after dissociation, the cultured population may have contained a mixture of neoplastic and non-neoplastic cells, which represents an additional limitation of the assay.

**Figure 3:**
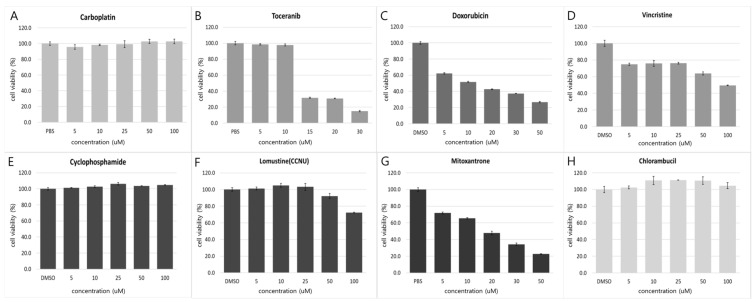
Ex vivo drug sensitivity testing results. Cell viability (%) assessed by CellTiter-Glo^®^ luminescence assay of surgically resected tumor–derived cells after exposure to the indicated anticancer agents at graded concentrations. Vehicle controls (PBS or DMSO) were included as indicated. Data are presented as mean ± SD from technical triplicates (*n* = 3). Tumor cells were exposed to eight chemotherapeutic agents: (**A**) carboplatin, (**B**) toceranib, (**C**) doxorubicin, (**D**) vincristine, (**E**) cyclophosphamide, (**F**) lomustine (CCNU), (**G**) mitoxantrone, and (**H**) chlorambucil. Consistent with the descriptive interpretation above, doxorubicin and mitoxantrone demonstrated concentration-dependent reductions in viability under the tested conditions. Toceranib and vincristine showed variable concentration-dependent effects, whereas carboplatin, cyclophosphamide, and chlorambucil exhibited minimal activity.

The detailed dose–response profiles are shown in Figure 3.

The selection of candidate drugs was primarily based on histopathological diagnosis and previously reported therapeutic approaches. The DST profile was considered as supplementary reference information during clinical deliberation, rather than a determinant of protocol selection.

## 3. Therapeutic Management

Because the tumor was distributed across multiple hepatic lobes, curative surgical resection was not feasible. Therefore, a stepwise chemotherapeutic strategy was developed with consideration of DST findings as supportive functional information. The therapeutic protocols applied during the course of treatment are summarized in Supplementary Table S1.

**Figure 4:**
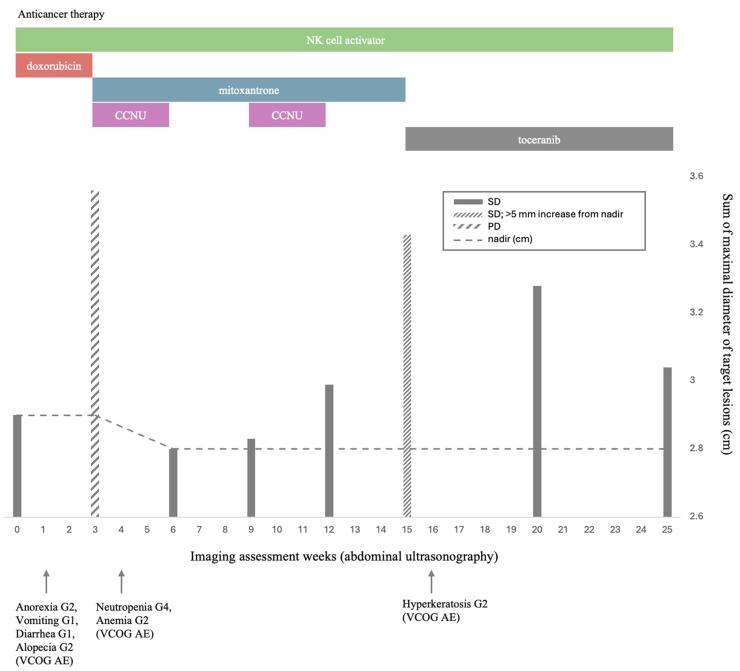
Timeline of therapeutic interventions, imaging-based tumor response, and adverse events. Bars represent the sum of maximal diameters of target lesions (SMDTL) measured at each ultrasonographic assessment time point in accordance with VCOG RECIST criteria. The dashed line indicates the nadir value. Patterned bars indicate response categories (SD, SD with >5 mm increase from nadir, and PD). The upper panel illustrates administered anticancer therapies over time. Arrows below the *x*-axis denote clinically significant adverse events.

Treatment response was assessed using abdominal ultrasonography. Two representative lesions—Mass 1, located in the lateral hepatic lobe, and Mass 2, positioned adjacent to the gallbladder—were designated as target lesions. The sum of the maximal diameters of these lesions (SMDTL) was used as the primary index for response evaluation (Figure 1 and Figure 4). Individual target lesion measurements, including maximal diameters of each hepatic mass and corresponding SMDTL calculations at each evaluation time point, are detailed in Supplementary Table S2. Baseline and follow-up measurements were recorded in accordance with VCOG RECIST criteria [6].

Non-target lesions were monitored qualitatively for interval enlargement or emergence of new lesions, and no additional measurable disease sites were identified during follow-up.

Longitudinal SMDTL measurements, serial laboratory parameters, and clinical events throughout the treatment course are summarized in Supplementary Table S2. Serial biochemical monitoring included alanine aminotransferase (ALT) and alkaline phosphatase (ALP), which demonstrated fluctuations during treatment but no sustained progressive deterioration consistent with hepatic failure. Detailed longitudinal laboratory values are provided in Supplementary Table S2. Aspartate aminotransferase (AST), gamma-glutamyl transferase (GGT), and serum bile acids were not consistently assessed during follow-up and represent a limitation of this report. Therefore, comprehensive longitudinal hepatic function assessment was limited. However, no clinical signs of hepatic failure (ascites, encephalopathy, hypoalbuminemia, coagulopathy) were observed during the monitored period. Figure 4 presents an integrated graphical overview of the overall therapeutic sequence, tumor responses, and key clinical outcomes. Anticancer therapy proceeded sequentially: doxorubicin administered concurrently with an NK cell activator; mitoxantrone plus lomustine (CCNU) with prednisolone and continued adjunct immunomodulatory therapy; and subsequently toceranib with continued adjunct immunomodulatory therapy. Because multiple agents were administered sequentially and, at times, concurrently, causal attribution of clinical response or failure to any single component is not possible; outcomes should be interpreted as reflecting combined treatment effects and real-world clinical decision-making.

Major adverse events occurred at various points during therapy, including anorexia (G2), vomiting (G1), diarrhea (G1), alopecia (G2), neutropenia (G4), anemia (G2), and hyperkeratosis (G2), graded according to VCOG-CTCAE criteria [7].

## 4. Follow-Up and Outcomes

### 4.1. First-Line Protocol and Clinical Course (Week 0–3)

During Cycle 1 (Week 0–3), doxorubicin was administered at a dose of 30 mg/m^2^, intravenously every 3 weeks, concomitantly with a cytokine-based NK cell activator (IL-12, IL-15, and IL-23) combined with sodium selenite, prepared as a 10-mL infusion delivered over 5 min. The infusion duration was selected for practical clinical administration and was not based on pharmacokinetic optimization studies. This regimen was selected as part of an adaptive clinical strategy, with DST findings considered as supportive information rather than a deterministic guide. No pharmacodynamic monitoring was performed to confirm systemic cytokine bioactivity.

Notable adverse events emerged early in treatment; by Day 3, Grade 3 anorexia, Grade 1 vomiting and diarrhea, and Grade 2 weight loss were recorded. At the Week 3 evaluation, the sum of the maximal diameters of the target lesions (SMDTL) increased from 2.90 cm at baseline to 3.56 cm (+22.8%), meeting the criteria for progressive disease (PD). Although this increase exceeded the RECIST PD threshold (≥20%), classification strictly followed VCOG RECIST criteria. Consequently, continuation of the doxorubicin-based regimen was deemed unlikely to provide additional clinical benefit.

### 4.2. Second-Line Protocol and Clinical Course (Week 3–15)

Following ultrasound-documented progression, the therapeutic regimen was switched to mitoxantrone (5.5 mg/m^2^ intravenously every 3 weeks) in combination with lomustine (70 mg/m^2^ orally every 6 weeks). The NK cell activator was maintained on a 3-week schedule as adjunct immunomodulatory therapy, although its biological activity and clinical contribution could not be independently confirmed. Prednisolone (PDS) was initiated at 1 mg/kg orally twice daily and tapered gradually in accordance with clinical stability.

During Cycle 1 of second-line treatment (Week 3–6), Grade 1 anorexia, Grade 4 neutropenia (0.35 K/µL), and Grade 2 anemia (HCT 30.5%; treated with darbepoetin) were observed. The patient remained clinically stable without fever or signs suggestive of sepsis. The neutropenic episode was afebrile and managed on an outpatient basis with a single dose of G-CSF under strict monitoring with scheduled complete blood count reassessment. Although hospitalization is generally recommended for Grade 4 neutropenia according to VCOG-CTCAE guidelines, the decision for outpatient management was made based on the absence of fever, preserved appetite, and strict owner compliance with monitoring instructions. The owner was instructed to seek immediate re-evaluation if any concerning signs developed. Given the absence of clinical complications, the treatment schedule was maintained as planned with close CBC monitoring. Despite hematologic toxicities, Week 4 imaging revealed a reduction in SMDTL to 2.84 cm (−2.1% from baseline), consistent with stable disease (SD).

Cycle 2–3 (Week 6–15) resulted in a nadir SMDTL of 2.80 cm, and the patient remained clinically stable except for persistent Grade 1 ALP elevation. PDS tapering progressed to 0.75 mg/kg twice daily, and subsequently to 0.5 mg/kg once daily, without clinical deterioration.

At week 15, prior to Cycle 5, SMDTL increased by 6.3 mm from the nadir and by +18.3% from baseline. Because this increase remained below the ≥20% threshold for progressive disease under VCOG RECIST criteria, the response category was maintained as stable disease; however, the upward trend supported closer surveillance. In addition, the owner was no longer able to continue regular hospital visits for injectable chemotherapy and strongly preferred an oral regimen; therefore, the protocol was electively transitioned to toceranib-based targeted therapy for feasibility and treatment continuity rather than due to RECIST-defined progression.

### 4.3. Third-Line Protocol and Clinical Course (Week 15–25)

Beginning at Week 15, toceranib phosphate (Palladia®) was initiated at 2.75 mg/kg orally every 48 h [8]. The cytokine-based NK cell activator was administered every 4 weeks as adjunct therapy.

Under this protocol, SMDTL remained stable with a mild decrease over time, and radiologic assessments through Week 25 confirmed maintenance of SD without evidence of further progression. The primary adverse event was Grade 2 hyperkeratosis, which was effectively controlled with topical therapy.

## 5. Discussion

Hepatic neuroendocrine carcinoma (NEC) in dogs is an exceedingly rare neoplasm [1,2], and most cases are diagnosed at an advanced stage, often accompanied by multifocal hepatic involvement or regional lymph node metastasis [2]. The prognosis is generally poor, with some reports describing rapid clinical deterioration shortly after diagnosis. To date, no standardized therapeutic regimen has been established. Although doxorubicin- or toceranib-based protocols have been described in isolated case reports, their clinical responses remain inconsistent and lack reproducibility [3,4]. Earlier veterinary reports used the term “carcinoid,” which has largely been replaced by neuroendocrine carcinoma terminology in contemporary pathology nomenclature.

In light of this therapeutic uncertainty and the absence of an evidence-based standard of care, we performed an ex vivo drug sensitivity test (DST) at the time of diagnosis to explore relative drug responsiveness as supportive information to complement clinical decision-making.

In the descriptive ex vivo assay, doxorubicin was associated with dose-dependent reductions in cell viability under the tested conditions. Mitoxantrone and toceranib demonstrated comparable concentration-dependent effects, whereas carboplatin, cyclophosphamide, and chlorambucil exhibited minimal activity. Although vincristine showed in vitro sensitivity, it was not incorporated into the treatment protocol due to limited supporting evidence in canine hepatic NEC and concerns regarding additive toxicity within an already intensive multimodal regimen. These observations are consistent with previously reported use of doxorubicin or toceranib in canine hepatic NEC [3,4].

Accordingly, doxorubicin was selected as the initial cytotoxic agent, with DST findings considered as supportive information during protocol selection. As an immunomodulatory approach during cytotoxic chemotherapy, a cytokine-based NK cell activator was incorporated as adjunct immunomodulatory therapy; however, immune activation was not directly assessed.

The first-line protocol consisted of doxorubicin (30 mg/m^2^ IV, q3wk) combined with the NK cell activator during the initial 3-week induction phase. However, restaging at week 3 revealed progressive disease (PD) based on RECIST criteria, accompanied by anorexia, weight loss, and deterioration in quality of life (QoL). Despite apparent ex vivo responsiveness in the DST assay, the tumor exhibited unexpectedly rapid in vivo progression, necessitating protocol modification. Consequently, treatment objectives were re-aligned from high-intensity cytotoxic exposure to a more sustainable, tolerable, and QoL-oriented therapeutic approach.

Following disease progression on the first-line regimen, repetition of similar cytotoxic agents solely based on DST sensitivity was deemed unlikely to confer additional benefit. Instead, oral agents demonstrating measurable DST responsiveness were considered alongside clinical feasibility, tolerability, and owner preference.

Lomustine (CCNU), despite its limited DST reactivity, was selected due to its oral formulation, manageable toxicity profile, and clinical utility across various solid tumors [9]. Prednisolone was included for its anti-inflammatory, anti-neoplastic, and appetite-stimulating effects, facilitating QoL maintenance.

Thus, the second-line protocol represented a balanced strategy incorporating DST outcomes, drug tolerability, dosing practicality, owner compliance, and real-world clinical logistics.

Importantly, the novelty of this report lies not in the therapeutic endpoint itself, but in the structured documentation of sequential decision-making and adaptive protocol modification in a rare canine hepatic NEC case without standardized treatment guidance.

Sustained stable disease (SD) was observed following transition to the third-line regimen comprising toceranib with continued adjunct immunomodulatory therapy; however, the independent contribution of each component cannot be determined. Toceranib was the only DST-sensitive agent suitable for prolonged oral administration, and its inhibition of VEGFR and PDGFR provided a rational mechanism for controlling tumor growth and angiogenesis. Literature also supports long-term survival exceeding 25 months with toceranib monotherapy in canine hepatic NEC [4], aligning with the treatment rationale applied here.

Although DST provided comparative functional reference information, it did not function as a definitive predictor of clinical efficacy. The discordance between strong ex vivo sensitivity to doxorubicin and rapid clinical progression underscores important limitations of monoculture-based drug sensitivity platforms. Ex vivo assays do not capture tumor–stromal interactions, vascular perfusion constraints, immune-mediated modulation, or intratumoral clonal diversity, all of which may substantially alter therapeutic response in vivo. Additionally, hepatic tumor burden and altered pharmacokinetics in the setting of extensive liver involvement may influence effective drug exposure. This discrepancy suggests that DST findings should be interpreted as functional hypothesis-generating observations rather than direct predictors of clinical efficacy.

Furthermore, short-term viability assays measure acute cytotoxicity rather than long-term clonogenic survival or tumor repopulation dynamics, which may partially explain the discrepancy between in vitro sensitivity and rapid in vivo progression. Such discordance reinforces the need to integrate functional assay results within dynamic clinical monitoring rather than relying on them in isolation.

Thus, DST should be interpreted as a functional adjunct, and the findings should be regarded as exploratory and hypothesis-generating rather than predictive or quantitatively validated. In this case, treatment decisions were continually refined using an adaptive model that incorporated imaging findings (RECIST), QoL metrics, hematologic parameters, and adverse event profiles. While this case does not represent precision oncology in a formal or validated sense, it reflects a pragmatic attempt to incorporate exploratory functional data into ongoing clinical decision-making. Recent advances in organoid-based assays and immune–stromal co-culture systems may overcome current limitations by better recapitulating in vivo biology [5,10].

IL-12 and IL-15 have been reported to enhance NK cell activation pathways in experimental systems [11], and IL-23 has been associated with modulation of IFN-γ–related responses [12]. Selenium has also been implicated in immune metabolic regulation within tumor microenvironments [13]. However, these observations are derived primarily from human or experimental models and cannot be directly extrapolated to clinical canine oncology without species-specific pharmacodynamic validation. No immune monitoring was performed in this case; therefore, the biological activity and independent clinical contribution of this adjunct regimen cannot be determined.

This single-patient report limits generalizability. Because multiple therapeutic agents were administered sequentially and in combination, the observed clinical benefit cannot be attributed to any single drug. The discrepancy between DST and clinical outcomes underscores that functional ex vivo testing currently serves as supportive evidence rather than a definitive therapeutic determinant. Furthermore, the absence of immune biomarker assessment restricts interpretation of the NK cell activator’s mechanistic contribution. The Grade 4 neutropenia observed during second-line chemotherapy was clinically asymptomatic and successfully managed with a single outpatient dose of G-CSF, allowing continuation of treatment while maintaining patient safety. Notably, no progressive biochemical evidence of hepatic decompensation was observed during the treatment course.

Nevertheless, this case demonstrates the feasibility of an adaptive therapeutic strategy driven by clinical response and tolerance, with DST serving as supportive information in a tumor type lacking established standards of care. Future multicenter investigations integrating DST with immune profiling—such as NK cell assays and tumor microenvironment analyses—may enable the development of more quantitatively validated treatment frameworks for rare cancers.

At the time of manuscript revision (1 February 2026), the patient remains alive 48 weeks after treatment initiation with sustained radiologic stability and preserved quality of life.

## 6. Conclusions

This case illustrates the application of an adaptive, clinically guided multimodal treatment strategy in a dog with advanced hepatic neuroendocrine carcinoma. An ex vivo drug sensitivity assay was performed as supportive, hypothesis-generating information to complement clinical decision-making rather than to dictate therapeutic selection. Despite early discordance between in vitro findings and in vivo response, sequential protocol adjustments based on imaging findings, tolerability, and quality-of-life considerations were associated with sustained clinical stability.

Because multimodal therapy was administered throughout the treatment course, the independent contribution of any single therapeutic component, including toceranib and adjunct immunomodulatory therapy, cannot be conclusively determined. Nevertheless, this case underscores the potential value of iterative, response-driven treatment refinement in rare canine malignancies, while highlighting the limitations of ex vivo assays and the need for further validation in prospective studies.

## Data Availability

The data generated and analyzed during this study are included in the published article and its Supplementary Materials. Additional information is available from the corresponding author upon reasonable request.

## Supplementary Materials

The following supporting information can be downloaded at: https://www.mdpi.com/article/10.3390/ani16040646/s1: Supplementary Figure S1: Gross appearance of the resected hepatic neuroendocrine carcinoma mass obtained during surgery; Supplementary Table S1: Summary of therapeutic protocols applied during the entire treatment course; Supplementary Table S2: Timeline of therapeutic interventions, tumor response, and serial laboratory monitoring.

## Abbreviations

The following abbreviations are used in this manuscript:

NEC	Neuroendocrine Carcinoma
DST	Drug Sensitivity Test
SD	Stable Disease
PD	Progressive Disease
SMDTL	Sum of Maximal Diameters of Target Lesions
TKI	Tyrosine Kinase Inhibitor
NK	Natural Killer
QoL	Quality of Life
RECIST	Response Evaluation Criteria in Solid Tumors
VCOG	Veterinary Cooperative Oncology Group
VCOG-CTCAE	Veterinary Cooperative Oncology Group–Common Terminology Criteria for Adverse Events
