# Supplementary material for: Adaptive, Clinically Guided Multimodal Therapy with Supportive Drug Sensitivity Testing in a Dog with Hepatic Neuroendocrine Carcinoma: A Case Report
Source: Animals (Basel). 2026 Feb 17;16(4):646. doi: 10.3390/ani16040646 (PMC12937194; doi:10.3390/ani16040646)

Supplementary Figure S1. Intraoperative findings and excised hepatic nodules.
(A) Partial hepatectomy was performed to obtain an excisional biopsy. Intraoperatively, well-demarcated round nodules were visible on the liver surface.
(B) Gross appearance of the excised nodules, approximately 1 cm in diameter, submitted for histopathological evaluation and drug sensitivity testing (DST).

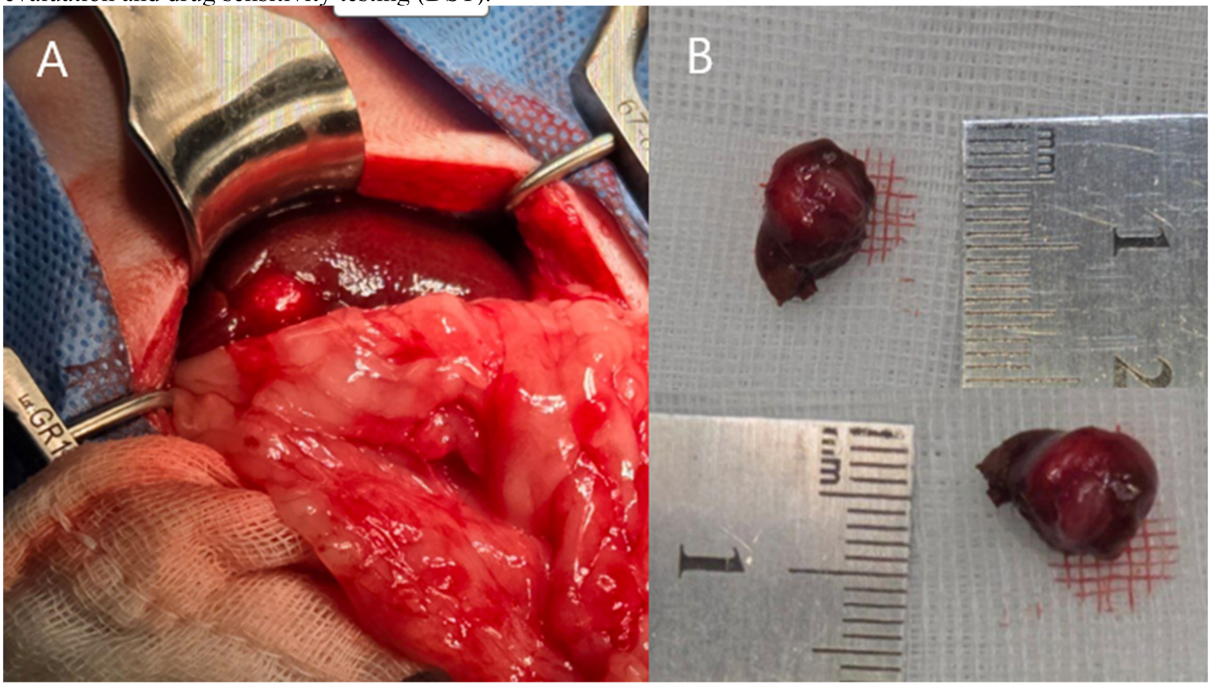

Supplement: Supplementary file 1 [file animals-16-00646-s001.zip › Supplementary_Figure_S1.pdf]
