# Supplementary material for: Adaptive, Clinically Guided Multimodal Therapy with Supportive Drug Sensitivity Testing in a Dog with Hepatic Neuroendocrine Carcinoma: A Case Report
Source: Animals (Basel). 2026 Feb 17;16(4):646. doi: 10.3390/ani16040646 (PMC12937194; doi:10.3390/ani16040646)
Supplement: Supplementary file 1 [file animals-16-00646-s001.zip › Supplementary_Table_S1, S2.pdf]

Supplementary Table S1. Summary of therapeutic protocols applied during the entire treatment course.

Protocol	Components	Dosage & Schedule	Comments
First-line	Doxorubicin + NK activator	Doxorubicin 30 mg/m² IV q3wk; NK activator (IL-12/IL-15/IL-23 + selenium)	High DST sensitivity but early PD
Second-line	Mitoxantrone + Lomustine (CCNU) + NK activator + PDS	Mitoxantrone 5.5 mg/m² IV q3wk; Lomustine 70 mg/m² PO q6wk; PDS tapering; NK activator every 3 wk	Achieved temporary SD
Third-line	Toceranib + NK activator	Toceranib 2.75 mg/kg PO q48h; NK activator every 4 wk	Durable SD to Week 25

Supplementary Table S2. Timeline of therapeutic interventions, tumor response, and serial laboratory monitoring.
This table summarizes treatment protocols, changes in tumor size, disease progression, and hematologic/biochemical parameters throughout the course of therapy. Tumor burden was assessed using the sum of maximal diameters of two representative lesions (SMDTL), with nadir values recorded for reference. Tumor response was classified according to VCOG RECIST criteria. Clinical adverse events (graded by VCOG-CTCAE) and therapeutic interventions are indicated in the timeline. Serial laboratory parameters include alanine aminotransferase (ALT), alkaline phosphatase (ALP), albumin (Alb), hematocrit (Hct), and neutrophil count (Neu).

Weeks	clinical event	mass			progression evaluation				ALT	ALP	Alb	Hct	Neu
		Max of mass 1 diameter(cm)	Max of mass 2 diameter(cm)	SMDTL (cm)	change rate(%) to baseline	nadir (cm)	SMDTL-nadir (cm)	stage	10 ~ 125 (U/L)	23 ~ 212 (U/L)	2.3 ~ 4 (g/dL)	37.3 ~ 61.7 (%)	2.95 ~ 11.64 (K/μL)
-5	First detection with US & CT		1.26						24	61	3.1	41.4	4.29
-3	Liver Trucut biopsy	1.51	1.17	2.68					UNDER	47		44	4.71
-1	Liver surgery biopsy & Chemosensitivity assay	1.56	1.4	2.96					15	44	3.4	39.5	4.9
**0**	**doxorubicin + NK activator → VCOG AE grade (anorexia G2, vomiting G1, diarrhea G1, alopecia G2)**	1.51	1.39	2.9	0.00%	2.9	0	-	11	50	3.3	42	7.68
3	PD conform; CCNU + Mitoxantrone + NK activator + PDS 1mpk BID	1.81	1.65	3.56	22.76%	2.9	0.66	PD	10	20	2.9	37	5.51
4	→ VCOG AE grade (neutropenia G4, anemia G2)	1.51	1.33	2.84	-2.07%		2.84					30.6	0.35
6	Mitoxantrone + NK activator	1.45	1.35	2.8	-3.45%	2.8	0	SD	53	271	3	25.9	9.32
9	CCNU + Mitoxantrone + NK activator	1.46	1.37	2.83	-2.41%	2.8	0.03	SD	111	370	2.7	37.8	13.72
12	Mitoxantrone + NK activator	1.62	1.37	2.99	3.10%	2.8	0.19	SD	76	477	2.6	34.7	11.16
15	SDTL increased but still SD; Toceranib TOD + NK activator → VCOG AE grade (Hyperkeratosis G2)	1.76	1.67	3.43	18.28%	2.8	0.63	SD*	16	111	2.6	41.6	6.18
20	Toceranib TOD + NK activator	1.68	1.6	3.28	13.10%	2.8	0.48	SD	12	55	2.1	39	4.54
25	Toceranib TOD + NK activator	1.49	1.55	3.04	4.83%	2.8	0.24	SD	UNDER	53	2.6	42.7	7.19
